# Loan default prediction of Chinese P2P market: a machine learning methodology

**DOI:** 10.1038/s41598-021-98361-6

**Published:** 2021-09-21

**Authors:** Junhui Xu, Zekai Lu, Ying Xie

**Affiliations:** Department of Sociology, School of Public Administration, Guangzhou University, Guangzhou, 510006 China

**Keywords:** Mathematics and computing, Computer science, Information technology

## Abstract

Repayment failures of borrowers have greatly affected the sustainable development of the peer-to-peer (P2P) lending industry. The latest literature reveals that existing risk evaluation systems may ignore important signals and risk factors affecting P2P repayment. In our study, we applied four machine learning methods (random forest (RF), extreme gradient boosting tree (XGBT), gradient boosting model (GBM), and neural network (NN)) to predict important factors affecting repayment by utilizing data from Renrendai.com in China from Thursday, January 1, 2015, to Tuesday, June 30, 2015. The results showed that borrowers who have passed video, mobile phone, job, residence or education level verification are more likely to default on loan repayment, whereas those who have passed identity and asset certification are less likely to default on loans. The accuracy and kappa value of the four methods all exceed 90%, and RF is superior to the other classification models. Our findings demonstrate important techniques for borrower screening by P2P companies and risk regulation by regulatory agencies. Our methodology and findings will help regulators, banks and creditors combat current financial disasters caused by the coronavirus disease 2019 (COVID-19) pandemic by addressing various financial risks and translating credit scoring improvements.

## Introduction

### Background

Financial enterprises operating on the internet are developing rapidly with the advent of the Web 3.0 era. Peer-to-peer (P2P) lending is an online financial platform designed to provide small and microloans among strangers. Zopa, the first P2P company, was set up in London in 2005 and was followed by high-speed growth of P2P lending companies worldwide. As a developing country, China has experienced a major boom in P2P lending over the past five years; at the same time, China is the leading market concerning both the total volume of loans and the number of borrowers. The earliest P2P company in China was created in 2006^1^. Five years later, 50 platforms were in operation, and the number jumped to 1931 as of December 2017, according to the statistics of WDZJ.com (a web loan information platform). As of June 2018, the volume of the P2P net loan industry was 175 billion and 723 million RMB, and the Chinese P2P lending market became the largest worldwide. However, Chinese internet-based financial institutions have accumulated substantial financial risks due to the prosperity of the P2P lending industry. By the end of June 2018, the number of ongoing P2P lending companies had dropped to 1836, 95 less than at the end of 2017. In the past 5 years, P2P lending companies in China have failed due to various problems, such as unreal investments, inability to repay loans, platform revocation, and juridical person escape, making P2P lending a potential disastrous venture full of potential financial risks.

### Problem statement

Given the above discussion, understanding the factors that lead to repayment failure is crucial and necessary for P2P lending platforms to perform borrower screening and for regulatory agencies to conduct risk management. A strong ability to predict the factors affecting repayment is of great importance to mitigate information asymmetry among borrowers, P2P lending platforms and lenders. Given that the existing assessments for P2P borrowers are inadequate and subject to unknown factors, a wide variety of personal information should be verified, and whether the information (income, job, identification, credit situation, etc.) should be displayed on the borrowing webpage. This change led us to the following question: Does borrower verification predict future repayment behavior? P2P lending companies review the borrower's information and give each borrower a score according to an evaluation method. Previous studies have focused on the importance of credit scores in predicting default. Our research emphasizes various borrower certifications, such as video, mobile phone, job, income, marital status, and residence.

P2P lending companies in China have undergone extensive reorganization. In the past few years, classic statistical analysis and mathematical programming have brought about the rapid development of several data mining technologies in the field of credit rating and the assessment of borrowers’ default risk. Due to the limitedness of specialists and ignorance of major signals, recent interest in machine learning to determine the driving factors of repayment failure has arisen in the field of P2P lending to improve the veracity and efficiency of decision-making concerning borrowers’ screening by platforms and risk supervision by regulators. Increasingly complicated machine learning methods have helped policymakers analyze a great amount of data promptly. Considering the importance and urgency of evaluating the growing risk of P2P borrowers, we endeavor to improve the predictive power by taking advantage of the latest machine learning methods.

### Problems of existing studies

Compared with the existing research, our paper contributes to the literature on the fast-expanding P2P lending industry in the following aspects. Despite several papers discussing performance and repayment failure in P2P lending, our research sheds light on predicting the default risk of P2P borrowers on Renrendai. First and foremost, our paper is the first study dedicated to forecasting the default risk of borrowers and examining the factors that determine the likelihood of default in the Chinese P2P platform by exploiting machine learning methods. Several articles on predicting P2P default are related to our research. For example, Yu and Zhu utilize data mining (DM) techniques to forecast the performance of P2P loans^2^. The authors compare five DM models with two measures, namely, average percent hit rate and area of the cumulative lift curve, to assess the prediction results. Huang used support vector machine (SVM) and neural network (NN) models to forecast credit ratings and concluded that the SVM model achieves slightly better prediction accuracy^3^. These two papers compare the predictive accuracy of various methods, whereas our study explores the factors relevant to borrowers’ repayment risk, which is ranked in order of their importance to default. Therefore, to the best of our knowledge, our paper is among the first to use machine learning methods to predict repayment failure. Second, our dataset is extracted from one of China's top 10 P2P companies and includes special data items captured by background network software. In addition to borrowers' loan information, credit history and demographic data, we were able to obtain all the authentication records of the platform for the borrowers, especially acquiring video and mobile phone authentication, which any other literature has not discussed according to our understanding. Third, our study found some apparently contradictory results, such as a higher default rate for borrowers who have passed mobile phone verification or video authentication. These conclusions provide a deeper understanding of the platform mechanism and borrowers’ information for a meaningful in-depth study. Furthermore, we should be very careful in explaining the conclusions of the empirical research. Fourth, our findings will be particularly useful for government regulation of P2P companies and help companies identify better borrowers to list on their websites.

### Gaps and contribution

As we stated in the problems of existing studies, although there have been many studies on the application of machine learning on P2P platforms, there are still relatively few studies on P2P prediction in mainland China. In fact, many studies on P2P prediction in mainland China use traditional statistical models such as logit and probit models. Our research fills this gap. Second, in existing research, the methods of obtaining data are obtained through questionnaires or web crawlers. The amount of data is relatively small, and the data quality is not high. Our research uses the first-hand data provided by Renrendai, which has more features and a larger amount of data, which also makes our conclusions more convincing. Finally, our research also fills the gap in the ignorance of the research on the importance of features in the previous literature. We used 4 models to obtain the importance of each feature and predicted the default factors of individuals.

### Methodology and findings

In this study, we used the loan default data provided by Renrendai to train our models. The data contain 54,477 observations and 28 features (including credit score, borrower certification level and whether the borrower has a car). We use the synthetic minority oversampling technique (SMOTE) as our sampling method to balance the dataset. Then, we trained 4 models on the dataset, namely, the gradient boosting model (GBM), NN, extreme gradient boosting tree (XGBT) and random forest (RF) models. The GBM and NN are used to obtain the importance of the characteristics that affect the default. We found that the establishment of an effective scoring mechanism for evaluating borrower credit can effectively help us identify the defaulter. After that, we used these four models to analyze the specific default further influencing factors of randomly selected individuals. Through this research, we found that individuals who are willing to provide personal information (unverified) tend to have a greater probability of default. In contrast, real asset information can significantly reduce the probability of default, and the amount of borrowing will affect the probability of default. Finally, we evaluated the performance metrics of the model through K-fold cross-validation and verified our evaluation results through t-tests and nonparametric methods (bootstrap method) in the “Discussion” section. We also compared some other studies. The results show that the random forest model performs well in the classification task of default, which can help internet finance companies effectively identify defaulters and adjust their strategies.

### Structure

First, we review the relevant literature on P2P lending from various perspectives, especially research related to default risk. Second, we present our dataset and introduce the relevant machine learning methods in “Data and method”. The results of the empirical research are revealed in “Empirical results and model comparison”. Finally, conclusions are drawn, and discussions are provided in “Discussion”.

## Related literature

According to the method employed, the literature on predicting default risk in P2P lending can be categorized into two classes. The first stream of research involves using traditional mathematical models, such as ordinary least squares (OLS), logit, probit, Cox, and Heckman models, to explore the possible factors affecting the probability of repayment failure. The second stream of research adopts machine learning or DM methods to predict the probability of repayment failure and compare various methods' strengths and weaknesses.

Based on data from different platforms, researchers have conducted a substantial quantity of work on the probability of successful fundraising, the interest rate of loans and the failure of repayment in the domain of P2P lending. Since our research uses machine learning methods to predict the factors that affect repayment failure, we subdivide the first stream of literature into four smaller categories according to the type of information.

### Category I

Predict default based on social relationships or the credit status of borrowers. Freedman and Jin were the first to study social relationships on P2P platforms^4^. Further study by Freedman and Jin based on a probit model shows that socially connected borrowers are always more likely to have their loans funded and more likely to default or be overdue^5^. Based on a unique dataset collected from social media, Ruyi Ge proves that self-disclosed social media information can be used to predict borrowers’ default probability^6,7^. These research findings contrast those of Lin and Viswanathan^8^. Lin extracted data from Prosper and reported the Cox model estimates of odds ratios^8^. They demonstrate that borrowers having a friendship with lenders are approximately 9% less likely to defer or repay unsuccessfully than those without. Xiangru Chen et al. find that the borrower’s group social capital reduces the default probability only if a group rating mechanism is implemented, and the number of group members of the borrower’s peers positively affects default probability when both a group leader reward and a group rating are appropriate^9^. Iyer et al. contrast the quality of the R2 from linear regression and a stylized receiver operating characteristic curve by examining Prosper data and find that lenders are 45% more accurate in predicting an individual’s likelihood of default on a loan than they are in forecasting a borrower’s precise credit score^10^.

### Category II

Predict default based on a borrower’s text description or photo display. Employing the Cox proportional hazard (CPH) model, Pope and Sydnor find significant racial disparities in P2P lending^11^. Compared to white borrowers, black borrowers have high default rates. A multinomial logistic model is utilized by Herzenstein et al. to show that borrowers with a larger number of identity claims in their narratives, especially about their success or trustworthiness, are less likely to repay their loans^12,13^. Extracting pictures of potential borrowers from the website of Prosper, Duarte runs a proportional, discrete-time hazard model of default and shows that seemingly trustworthy borrowers indeed default less often^14^. Similar to Pope and Sydnor’s findings^11^, Ravina finds that more attractive borrowers and those with a positive appearance tend to default less frequently^15^. However, Gonzalez and Loureiro argue that applicants who appear to be more attractive have default rates similar to those of ordinary-looking borrowers^16^. Liao et al. construct a probit model that indicates that a longer loan description is correlated with improved repayment and less default^17–19^. Dorfleitner uses IV probit regression to conduct an empirical study on the two most concerned European P2P platforms and arrives at conclusions that contradict those of Liao^20,21^. Spelling mistakes, text length and the presence of social and emotional keywords in the descriptive text are proven to be independent of default probability.

### Category III

Predict default based on platform mechanisms, such as guarantees, verifications, pricing policies or loan histories. Chen et al. used logit regression to predict the impact of all the information on a Chinese P2P lending platform^22^. They argue that the borrower’s credit status, living conditions, region of residence, personal income, number of successful borrowing attempts and number of on-time repayments negatively influence a borrower’s default probability, whereas the number of overdue payments, years of education, loan interest rate, and number of ahead-of-schedule payments positively influence a borrower’s overdue rate. Wei and Lin assess the different pricing mechanisms of Prosper.com by means of CPH estimates and find that the default rate under platform-mandated posted prices is higher than that in auctions^23^. The latest research by Xiang Hong-yu indicates that, overall, the higher the interest rate is, the more likely that the P2P lending platform will become problematic^24^. When the interest rates are especially high, interest rate and risk are positively correlated.

### Category IV

Predict default based on the demographic characteristics of borrowers. Guo Feng et al. use a probit model to conclude that married people have lower overdue repayment rates^25^. Li et al. reveal that more educated borrowers have lower default rates based on a probit model^18^. Tao et al. exploit logit regression to examine the influence of academic qualifications, and their results indicate that borrowers with more education have a higher default probability, which contradicts the results of Li^26^. To investigate the potential gender discrimination in Chinese P2P loans, Dongyu Chen et al. established a CPH model of the loan default rate. They report those female borrowers have lower default rates than male borrowers^27^.

In the above four parts, we review and sort the literature that uses traditional methods to predict repayment default in P2P lending. Machine learning methods are an alternative and increasingly popular research topic. Indeed, many DM techniques, including NNs, RFs^28^, decision trees (DTs) and SVMs, are commonly adopted to forecast repayment failure in P2P lending. NNs are employed by Odom^29^ and Tam^30^ to predict bank default and business bankruptcy. Many studies focus on the merits and demerits of machine learning methods in P2P default forecasting. Malekipirbazari and Aksakalli utilize a series of machine learning techniques^31^, such as RF, logistic regression, k-nearest neighbor, and SVM, to classify good and bad loans. They conclude that RF is more effective than Fair Isaac Corporation (FICO) and LendingClub grades in predicting defaults. Ajay et al.^32^ show that an NN-based credit scoring model is effective in default screening applications in P2P lending. Further empirical research by Vinod Kumar et al. confirms that an RF model performs well in identifying defaults, whereas DT is more powerful in finding borrowers with good credit^33^. Fu proposes a method that combines RF and NN for predicting borrowers’ status^34^. The combined method outperforms the LendingClub good borrower grades. By analyzing five credit databases, Chi et al. modernized credit prediction modelling and proved that multilayer perception (MLP)-based artificial neural networks (ANNs) are prominent in the classification of credit information and improving credit prediction^35^. Cui et al. built a novel filter-based feature selection model that uses graph-based features and exploits a steady-state random walk to encapsulate the major features of graph-based characteristics^36^. Their scientific experiments prove the effectiveness and practicability of the selection algorithm for predicting the default of P2P borrowers in China. Arturo Ramirez^37^ focuses on two approaches to analyzing default on the LendingClub platform. He regards loan default as a binary label problem and discusses it with the method of survival analysis. Yang developed an AdaBoost-SVM algorithm, and their experiment showed that the AdaBoost algorithm improves the classification accuracy of P2P lending platform risk^38^. The classification error is controlled within 5%. Applying six credit datasets, Abedin et al. proposed the application of support vector machine (SVM) and probabilistic neural network (PNN)-based CDP algorithms in predicting credit default, and they found that the PNN model is more robust than others^39^. Wang et al. collected information from text descriptions, social networks and macroeconomic data and developed methods to grab features from unstructured data to show that better P2P default risk prediction performance is achieved by combining information from different data sources^40^. RF and random survival forests are utilized individually by Wang et al. to predict the repayment failure of the borrower and the time to default^41^. Compared with the mixture cure model, CPH model and logistic regression, the proposed ensemble mixture RF has good performance with respect to predicting the monthly dynamic probability of default. Zhou Li demonstrated that credit risk evaluation models of P2P lending platforms based upon gradient boosting decision trees (GBDTs) and SVMs have higher accuracy and stability in prediction^42^. Using 3111 Chinese small business datasets, Mohammad Zoynul Abedin et al. combined a feature selection algorithm and classification algorithm and performed an experiment with 4 neural network (NN) models. They proved that artificial neural network (ANN) models are prevalent in predicting credit default^43^. Li et al. established a multiround ensemble learning model based on heterogeneous ensemble frameworks to predict repayment failure on a P2P lending platform in China^44^. In their model, the XGBoost, deep neural network and logistic regression are all treated as heterogeneous individual learners that go through a linear weighted fusion. In addition, the results indicate that the model has better predictive accuracy than traditional machine learning models and ensemble learning models. Zhao et al. present a visual analytic technique that determines and analyzes risks in P2P lending transactions^45^. Namvar et al.^46^ use machine learning techniques on LendingClub to extract important information and forecast whether a customer will be able to repay a loan. His thesis describes exploratory data analysis and feature engineering processes. Abedin et al. assessed the risk of financial decision support systems (FDSSs) and applied MLPs and SVMs in credit scoring and bankruptcy prediction. They confirmed that MLP5‐5 and MLP4‐4 are practicable topologies for the MLP algorithm, and the linear kernel function-trained SVM has better performance in prediction^47^.

Although an enormous number of studies have explored the merits and demerits of various methods, few have investigated the factors influencing P2P lending default using machine learning methods. Emekter et al.’s paper is the earliest study to implement binary logistic regression to examine default risk^48^. The results prove that credit rating, debt-to-income ratio, FICO score and revolving credit line utilization play an important role in loan default. The CPH test confirms that the probability of loan default increases with escalating credit risk of the borrower. Yu Jin and Yudan Zhu compared five DM models^2^: two DTs, two NNs and one SVM. The empirical results show that the loan term, annual income, loan amount, debt-to-income ratio, credit grade and revolving credit line utilization play important roles in loan default. Yuejin Zhang et al. constructed a credit scoring model based on a DT by fusing social media information^49^. They conclude that information about loans, social media and credit status are the most important factors for predicting repayment default. Xiaojiao Yu built an RF model and an XGBoost model that indicated external data^50^, such as the Zhima score (a credit score provided by Alipay), multiplatform stacking loan information, and social network information, were important factors in predicting overdue repayment of Chinese P2P funding. By studying unique clickstream data from one of the top 10 P2P platforms in China, Yang et al. find that the risk of a user’s upcoming loan is highly correlated with his repayment history and the sequence of his recent financial activities, i.e., his cash flow^51^. They propose deep credit to automatically acquire credit risk information based on the sequence of activities.

Different from the existing literature, our research focuses on the verification status of the platform for the borrower's mobile phone, video, job, income, marriage and other nonmandatory information and then uses five machine learning methods to test the helpfulness of the verifications in predicting borrowers’ repayment ability. Some validation information is not displayed on the website but is captured by computer software. Therefore, our data are more unique compared with the existing study and cover more complete information about borrowers. To the best of our knowledge, no paper has combined machine learning methods and verification status information for predicting repayment probability. Therefore, our research is an important supplement to the existing research.

## Data and method

### Data

Our research data are derived from Renrendai.com, one of the ten largest P2P lending platforms in China. The data for our empirical analysis span from Thursday, January 1, 2015, to Tuesday, June 30, 2015. The loan information provided by Renrendai can be classified into four parts. Part 1 contains descriptive information about the loan, such as the loan type (online verified, onsite verified or guaranteed by a designated company), loan amount, loan term, interest rate, time for successful funding, number of bidders, prepayment rate and method of repayment. During our data period, Renrendai has three methods of information authentication: (1) online verification by the P2P company; (2) onsite certification by the P2P company and (3) guarantee by a designated insurance company. Part 2 contains borrowers’ information and is subdivided into smaller categories. The first category provides borrowers' demographic information and credit grade, including nickname, age, educational background, marital status, and credit ratings. The second category is composed of the borrower’s records of repayment on Renrendai.com. The third category displays personal property, such as home or car ownership and home or car loans. The fourth category provides the borrower’s occupation description, including the borrower’s company, length of employment and work experience. Part 3 displays the verification status, including verification of the borrower’s video, mobile phone, job, income, marital status, assets, residence, credit report, education and Weibo. Part 4 describes the borrowers' current situation and the purpose of borrowing in a short paragraph. Tables 1 and 2 shows the description of our dataset.

**Table 1:** Description of the main variables.

Variable	Definition/indicator value
Default	Failure to repay a loan
Credit	Credit grade classified by Renrendai. Grades AA or A = 6, Grades B, C, D, E and HR are 5, 4, 3, 2, 1, respectively
Xyrz	Dummy variable, Renrendai has verified the borrower’s information only online, Xyrz = 1; otherwise = 0
Jgdb	Dummy Variable, Loan has been guaranteed by a company = 1; otherwise = 0
Sp	Dummy Variable, Renrendai has verified the borrower’s video data = 1; otherwise = 0
Sj	Dummy Variable, Renrendai has verified the borrower’s mobile phone = 1; otherwise = 0
Gz	Dummy Variable,Renrendai has verified the borrower’s job = 1; otherwise = 0
Sr	Dummy Variable,Renrendai has verified the borrower’s income = 1; otherwise = 0
Hy	Dummy Variable, Renrendai has verified the borrower’s marital status = 1; otherwise 0
Jzdzm	Dummy Variable, Renrendai has verified the borrower’s residence = 1; otherwise = 0
Xybg	Dummy Variable, Renrendai has verified the borrower’s credit report = 1; otherwise = 0
Xl	Dummy Variable, Renrendai has verified the borrower’s education level = 1; otherwise = 0
Wb	Dummy Variable, Renrendai has verified borrower’s microblog = 1; otherwise = 0
Jrrc	Number of bidders in a project
Nll	Interest rate on the loan
Bdze	Loan amount borrowed
Tqhkl	Cost of early repayment, rate is 1% or 0 (no charge)
Education	Education level of borrower: high school or below = 1; junior college = 2; undergraduate = 3; graduate = 4
Marriage	Dummy Variable, Marital status: borrower is married = 1; otherwise = 0
Income	Borrower’s income by month in RMB: less than 1,000 = 1; 1,000 − 2,000 = 2; 2,000 − 5,000 = 3; 5,000 − 10,000 = 4; 10,000 − 20,000 = 5; 20,000 − 50,000 = 6; more than 50,000 = 7
Position	Borrower’s job titles: ordinary worker = 1; junior manager = 2; senior manager = 3, top manager or firm owner = 4
Car	Dummy Variable, Borrower possess a car = 1; otherwise = 0
House loan	Dummy Variable, Borrower take on a home loan = 1; otherwise = 0
Car loan	Dummy Variable, Borrower take on a car loan = 1; otherwise = 0
Size	Borrower’s company size by employed population: Less than 10 = 1; 10 − 100 = 2; 100 − 500 = 3; 500-plus = 4
Region	Borrower’s city of residence. Cities are divided into five levels and take on values between 1 (low) and 5 (high). The value is calculated using an index based on economy, education, GDP, top 500 companies’ locations, per capita income, and airport proximity. The method of division is from www.360doc.com/content/15/0508/16/7785337_469017676.shtml

**Table 2:** Descriptive statistics of main variables.

Variable	obs	mean	min	max	Std. Dev
Default	54,477	0.0167	0	1	0.1281
Credit	54,477	5.5257	1	7	1.3604
Sdrz	54,477	0.7734	0	1	0.4186
Jgdb	54,477	0.1143	0	1	0.3181
Sp	54,477	0.0045	0	1	0.0668
Sj	54,477	0.0138	0	1	0.1167
Gz	54,477	0.9935	0	1	0.0806
Sr	54,477	0.9857	0	1	0.1186
Hy	54,477	0.0152	0	1	0.1224
Zc	54,477	0.0044	0	1	0.0659
Jzdzm	54,477	0.0133	0	1	0.1145
Xybg	54,477	0.9876	0	1	0.1105
Xl	54,477	0.0183	0	1	0.1340
Wb	54,477	0.0091	0	1	0.0950
Jrrc	54,477	51.3391	2	887	46.4629
Nll	54,477	11.9166	7	20	0.8453
Bdze	54,477	61,012	3000	480,000	31,931
Hkqx	54,477	27.3719	3	36	9.6374
Education	54,477	1.9859	1	4	0.7313
Jdrnl	54,477	36.6590	21	63	8.3711
Marriage	54,477	0.7101	0	1	0.4537
Income	54,477	4.3630	1	7	1.1531
House	54,477	0.6030	0	1	0.4893
Car	54,477	0.3205	0	1	0.4667
House loan	54,477	0.4419	0	1	0.4966
Car loan	54,477	0.0946	0	1	0.2928
Size	54,477	0.0810	0	1	0.2729
Region	54,477	4.0949	1	5	1.0193

### Method

Machine learning has made considerable achievements in recent years. The full potential of machine learning has yet to be realized, especially in many fields of risk forecasting related to credit loans from financial institutions and P2P lending on the internet. In our paper, six experiments are conducted with different machine learning methods. The first experiment aims to predict all possible factors that affect repayment. The factors are ranked in order of their importance. The next few experiments verify the impact of the factors on repayment default by using different methods. Each method is applied to the Renrendai.com data collected by Aijie Technology Company to compare their advantages and disadvantages, and the prediction accuracy/performance of all the methods is calculated and compared. We introduce the machine learning methods utilized in this study for better illustrating our research.

### SMOTE

To transform an imbalanced dataset into a balanced dataset. Our dataset is a typical imbalanced dataset where the default rate is much lower than the successful repayment rate. Machine learning algorithms applied to imbalanced classification datasets can produce biased predictions with misleading accuracies. We use the synthetic minority oversampling technique (SMOTE), which is a widely adopted approach, to address the class imbalance dataset. SMOTE uses bootstrapping and k-nearest neighbors to construct new minority class instances by transforming data based on feature space (rather than data space) similarities from minority samples^52,53^. SMOTE performs a combination of oversampling and undersampling to construct a balanced dataset.

**Figure 1:**
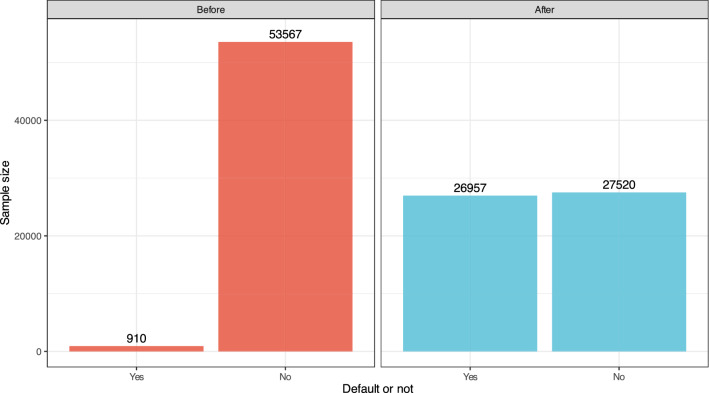
Transformation of an imbalanced dataset into a balanced dataset by using SMOTE.

As shown in Fig. 1, we successfully generate a balanced dataset using SMOTE.

### Gradient Boosting Model (GBM)

GBM constructs a prediction model based on an ensemble of weak prediction trees^54,55^. In this paper, we use the gradient boosting regression tree (GBRT) as follows:

1 $$ \begin{array}{*{20}c} {F\left( {\text{x}} \right) = \mathop \sum \limits_{{{\text{m}} = 1}}^{{\text{M}}} {\upgamma }_{{\text{m}}} {\text{h}}_{{\text{m}}} \left( {\text{x}} \right)} \\ \end{array} $$

where $h_{m} \left( x \right)$ are the weak learners in the context of boosting. Then, the GBRT builds the ensemble model in a forward function:

2 $$ \begin{array}{*{20}c} {F_{m} \left( x \right) = F_{m - 1} \left( x \right) + \gamma_{m} h_{m} \left( x \right)} \\ \end{array} $$

In each stage, a decision tree $h_{m} \left( x \right)$ is selected to minify the loss function $L$ given the present model $F_{m - 1}$ and its fit $F_{m - 1} \left( {x_{i} } \right)$:

3 $$ \begin{array}{*{20}c} {F_{m} \left( x \right) = F_{m - 1} \left( x \right) + \mathop {\min }\limits_{h} \mathop \sum \limits_{i = 1}^{n} L\left( {y_{i} ,F_{m - 1} \left( {x_{i} } \right) - h\left( x \right)} \right)} \\ \end{array} $$

This minimization problem is processed via the steepest descent direction, which can be calculated as follows:

4 $$ \begin{array}{*{20}c} {F_{m} (x) = F_{m - 1} (x) + \gamma_{m} \sum\limits_{i = 1}^{n} {\nabla_{F} L\left( {y_{i} ,F_{m - 1} \left( {x_{i} } \right)} \right)} } \\ \end{array} $$

### Neural network (NN)

Artificial neural networks are prediction models based on a simple mathematical process that mimics the functioning of a human brain. These networks allow for complex nonlinear relationships between a response variable and its predictors^56^. The process is defined as follows.

Given training examples $\left( {x_{1} , y_{1} } \right),\left( {x_{1} , y_{1} } \right), \ldots ,\left( {x_{n} , y_{n} } \right)$, where $x_{i} \in R^{n}$ and $y_{i} \in \left\{ {0,} \right. \left. 1 \right\}$, a multilayer perceptron (MLP) learns the function $f\left( x \right) = W_{2} g(W_{1}^{T} x + b_{1} )b_{2}$, where $W_{1} \in R^{m}$ and $W_{2} ,b_{1} , b_{2} \in R$ are model parameters. $W_{1} ,W_{2}$ represent the weights of the input and hidden layers, respectively, and $b_{1} ,b_{2}$ represent the bias added to the hidden layer and the output layer, respectively. $g\left( \cdot \right):R \to R$ is the activation function, which is the hyperbolic tangent function by default:

5 $$ \begin{array}{*{20}c} {g\left( z \right) = \frac{{e^{z} - e^{ - z} }}{{ e^{z} + e^{ - z} }}} \\ \end{array} $$

For binary tasks, MLP uses the cross-entropy loss function given as

6 $$ \begin{array}{*{20}c} {Loss\left( {\hat{y},y,W} \right) = - y\ln \hat{y} - \left( {1 - y} \right)\ln \left( {1 - \hat{y}} \right) + \alpha \left\| {\text{W}} \right\|_{2}^{2} } \\ \end{array} $$

where $\alpha \left\| {\text{W}} \right\|_{2}^{2}$ is an L2-regularization penalty that penalizes MLP models and $\alpha > 0$ is a nonnegative hyperparameter.

The algorithm stops when a given maximum number of iterations is reached or when the improvement in loss approaches a certain threshold.

### Random Forest (RF)

Given a training part, X = {x1..., xn} with responses Y = {y1..., yn}, random samples are selected (B times) with replacement to train decision trees

Then, the prediction for unseen sample x' is obtained by averaging the predictions from all the trained individual decision trees on x':

7 $$ \begin{array}{*{20}c} {\hat{f} = \frac{1}{B}\mathop \sum \limits_{b = 1}^{B} f_{b} \left( {x^{\prime}} \right)} \\ \end{array} $$

### Extreme Gradient Boosting Tree (XGBT)

XGBoost is an open-source software library that provides an innovative gradient boosting algorithm for C++, Java, Python, R, and Julia, which is an efficient implementation of the gradient boosting framework from Chen & Guestrin^57^.

### Factor importance & model metrics

Factor importance assesses the relative importance of that feature according to the predicting contribution of the target variable^58^. Referring to the research by Jain et al. on machine learning in customer sentiment analysis^59,60^, we used four metrics to evaluate algorithmic performance: Accuracy, Precision, Recall (also known as sensitivity), and F-Measure ($F_{1}$ score). On top of that, we have added Kappa metric^61^.They are defined as follows

8 $$ \begin{array}{*{20}c} {{\text{Accuracy }} = \frac{{{\text{TP}} + {\text{TN}}}}{{{\text{TP}} + {\text{TN}} + {\text{FP}} + {\text{FN}}}}} \\ \end{array} $$

where TP is true positive, TN is true negative, FP is false positive and FN is false negative.

9 $$ \begin{array}{*{20}c} {Kappa = \frac{{p_{o} - p_{e} }}{{1 - p_{e} }}} \\ \end{array} $$

where $p_{o}$ is the observed agreement and $p_{e}$ is the expected agreement. It calculates the predictive improvement over the performance of a classifier that simply guesses at random.

10 $$ \begin{array}{*{20}c} {Precision = \frac{TP}{{TP + FP}}} \\ \end{array} $$

As we mentioned in the *Accuracy* definition, TP is true positive, TN is true negative and FP is false positive. This indicates how many of the samples predicted to be positive are true positive samples.

11 $$ \begin{array}{*{20}c} {Recall = \frac{TP}{{TP + FN}}} \\ \end{array} $$

In this formula, TP is a true positive, TN is a true negative and FP is a false negative. In binary classification, *recall* is also called *sensitivity*. This indicates how many positive examples in the sample are predicted correctly.

The last metric is the $F_{1}$ score. The $F_{1}$ score is the harmonic mean of the *precision* and *recall*.

12 $$ \begin{array}{*{20}c} {F_{1} = \frac{2}{{Recall ^{ - 1} + Precision ^{ - 1} }} = 2 \cdot \frac{ Precision \times Recall }{{ Precision + Recall }} = \frac{ TP}{{TP + \frac{1}{2}\left( {FP + FN} \right)}}} \\ \end{array} $$

We use fivefold cross-validation, or out-of-sample testing techniques, to estimate the metrics of each predictive model. Cross-validation divides the data into a given number of subsets, performs the algorithm on the training dataset and then validates the performance on the testing dataset. The validation results are averaged over five rounds to provide an estimate of the model’s predictive performance.

## Empirical results and model comparison

### Empirical results

**Figure 2:**
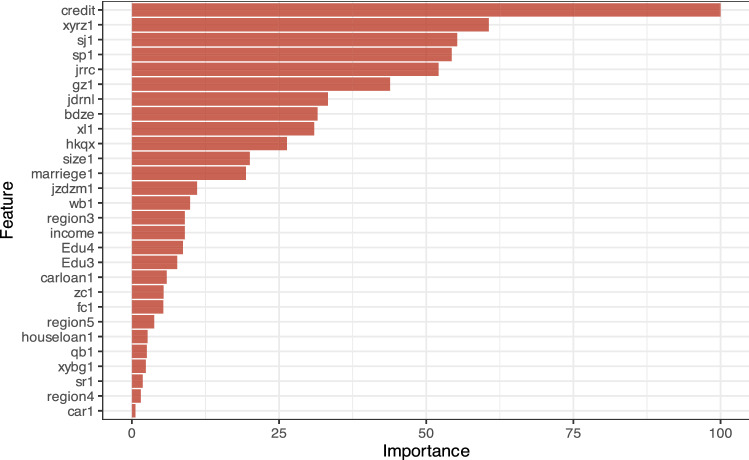
Importance ranking of the impact of variables on repayment failure (GBM).

We perform a preliminary prediction with GBM to assess the important variables that impact loan repayment failure. Figure 2 indicates that the five most important factors are as follows: credit rating, online credit authentication, mobile phone verification, video verification, and number of winning bidders in a project. The next five most important factors are borrower job authentication, age of the borrower, loan amount, education level of the borrower and loan term. Clearly, a borrower’s credit rating has the strongest influence on repayment failure, which means that the credit rating classifications of Renrendai are valuable in predicting a borrower’s repayment ability. The online certification of a borrower also has a substantial impact on borrower default. These conclusions have been verified by previous empirical studies^22^. Unexpectedly, cell phone verification and video authentication are vital to predicting borrower default. Since the forecasted results do not indicate which factors positively or negatively impact repayment, we continue with a more in-depth study.

**Figure 3:**
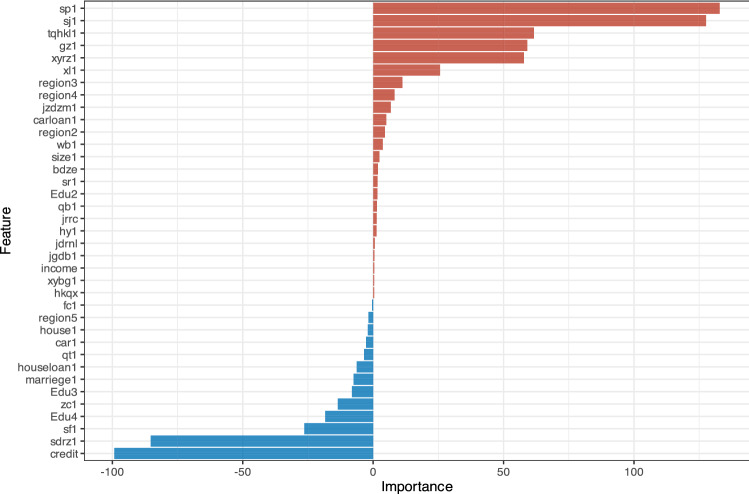
The importance sequence of variables that influence repayment failure (NN).

Further research is needed to determine whether the variables identified by GBM positively or negatively affect repayment failure. NN is implemented for further prediction. With 0 as the boundary, the right half of Fig. 3 shows the variables with positive effects, while the left half shows the variables with negative effects. Borrowers who have cell phone authentication or video authentication are more likely to fail in repayment, which is consistent with the prediction of the above GBM method. Similarly, borrowers with job authentication or online verification are more likely to delay repayment or completely fail to repay, which is consistent with the GBM predictions. Interestingly, repayment failure is more likely for borrowers who are charged for early repayment. In other words, if borrowers are not encouraged to make early payments, they are more likely to delay repayment. This conclusion is reasonable and is close to reality. Variables on the left half of the chart are negatively related to default, which means that these variables inhibit default. Borrower credit status, verification of a borrower’s identity or assets, and onsite verification reduce the risk of repayment failure. Furthermore, borrowers with a bachelor's degree or above are less likely to postpone repayment.

On the basis of these two tests, we can preliminarily state that verification of a borrower’s video, mobile phone, job and education level has a positive impact on repayment default. In contrast, authentication of a borrower’s identification and assets can reduce default behavior to a certain extent. These initial results arouse our interest. Does the verification Renrendai.com perform on borrowers predict future defaults? In the next sections, we will assess the verification processes via machine learning methods.

**Figure 4:**
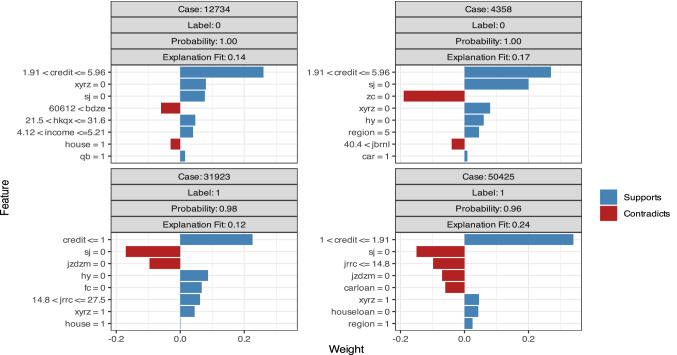
Variable weights of the GBM model.

Figure 4 and Table 3 show the variable weights calculated by GBM. “Label = 1” indicates repayment failure, whereas “Label = 0” indicates repayment success. The bar diagrams on the right half support the “Label”, whereas those on the left contradict the “Label”. The diagram shows that the credit score is vital to forecasting the probability of repayment success. Renrendai.com assigns credit scores according to the borrower’s personal information and borrowing history. When the credit score is greater than 1.91, the probability of successful repayment is high, and the probability of repayment failure increases when the credit score drops below 1.91, especially below 1. This result is similar to the previous experimental conclusions. Projects without borrowers’ mobile phone authentication or residence verification are less likely to result in repayment failure; that is, borrowers who voluntarily provide a cell phone or residence for verification are more likely to fail to repay their loan. This prediction is interesting and reasonable; we can infer that borrowers with good credit are not willing to spend time or money on phone number verification or residence authentication since these processes are not mandatory. Borrowers whose credit situation is not goodwill try harder to pass the P2P company’s examination. This additional effort implies poor credit, which leads to repayment failure. The upper right of Fig. 4 indicates that projects without asset verification have a higher probability of repayment failure, which means that personal assets can be regarded as a mortgage or guaranty and are basic requirements for evaluating a borrower’s repayment ability. Figure 4 also shows that the probability of repayment failure increases rapidly when the loan amount is larger than 60,612 or when more than 15 lenders support a project. This result is somewhat unexpected; however, considering that good projects would attract many investors and obtain the full complement of money in a relatively short period, this conclusion is rational. Generally, the assessment of GBM on the weights of multiple influencing factors indicates that borrowers without mobile phone verification or residence certification are more likely to repay successfully and that borrowers without asset authentication are more likely to default.

**Table 3:** Variable weights of the GBM model.

Questions	Prediction weight			
	Predicting success		Predicting failure	
	Label = 0	Label = 1	Label = 0	Label = 1
Credit grade classified by Renrendai	+ (1.91,5.96]	+ (1,1.91]	+ (1.91,5.96]	+ (0,1]
Whether Renrendai has verified the borrower's information only online	+ 0	+ 1	+ 0	+ 1
Whether Renrendal has confirmed the borrower's mobile phone	+ 0	− 0	+ 0	− 0
Whether Renrendal has confirmed the borrower's marriage	+ 0			+ 0
Whether Renrendal has confirmed the borrower's assets	− 0			
Whether Renrendai has verified the borrower's residence		− 0		− 0
Whether Renrendai has verified the borrower's education		− (0,14.8]		+ (14.8,27.5]
Number of bidders in a project				
Loan amount borrowed			− (60,612,	
Time for repayment, in months			+ (21.5,31.6]	
Borrower’s age	+ (40.4,100]			
Borrower’s income by month in RMB			+ (4.12,5.21]	
Whether borrower possess a house			− 1	
Whether borrower possesses a car	+ 1			
Whether borrower takes on a home loan		+ 0		
Whether borrower take on a car loan		− 0		
Borrower’s city of residence	+ 5	+ 1		
qb			+ 1	
fc				+ 0

Label = 0 means Repayments Success.

Label = 1 means Repayments Failure.

‘+’ means Supports, ‘−’ means contradicts.

**Figure 5:**
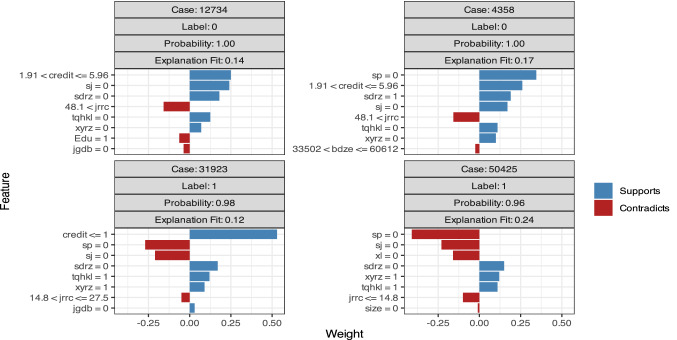
Variable weights of the NN model.

As we can see in Fig. 5 and Table 4.The analysis of NN model confirms some of our prediction results in GBM model. The credit score has the strongest positive effect on successful loan repayment. Borrowers with a credit score less than 1 have a higher probability of failing to repay, whereas borrowers with a credit score above 1.91 are more likely to repay on time. Both video authentication and mobile phone verification negatively affect repayment, suggesting that borrowers provide video or a mobile phone for verification when they are not competitive in applying for a loan or a P2P company does not approve them due to their credit. Borrowers with poor credit are predicted to fail with a higher probability. The method of information authentication also has an impact on repayment. Three methods of information authentication are accepted by Renrendai.com: onsite verification, online confirmation and guarantee by a designated company. The possibility of default is greater when a borrower’s information is not verified onsite, which means that the reliability of the onsite certification is the highest. Although online credit verification saves time, it is not as effective or accurate as onsite authentication in forecasting repayment default. Consistent with the previous result, NN indicates that a project is more likely to fail when supported by more than 48 lenders. Considering risk and return, investors are willing to invest only small amounts in projects with lower credit ratings, resulting in an increase in the number of shares in such a project. In addition, projects with the support of more lenders, especially more than 48 lenders, are more likely to fail. NN confirms that verification of a borrower’s video, mobile phone or education level is positively related to repayment failure, whereas onsite information authentication is negatively related to repayment failure in Chinese P2P lending.

**Table 4:** Variable weights of the NN model.

Questions	Prediction weight			
	Predicting success		Predicting failure	
	Label = 0	Label = 1	Label = 0	Label = 1
Credit grade classified by Renrendai	+ (1.91,5.96]		+ (1.91,5.96]	+ (0,1)
Whether Renrendai has verified the borrower's information only online	+ 0	+ 1	+ 0	+ 1
Whether Renrendal has confirmed the borrower's information onsite	+ 1		+ 1	+ 0
Whether loan has been guaranteed by a company			− 0	+ 0
Whether Renrendai has verified the borrower's video data	+ 0	− 0		− 0
Whether Renrendai has verified the borrower' s mobile phone	+ 0	− 0		− 0
Whether Renrendai has verified the borrower' s education		− 0		
Number of bidders in a project	− (48.1)	− (0,14.8]	− (48.1)	+ (14.8,27.5)
Interset rate on the load				
Loan amount borrowed	− (33,502;60,612]			
Cost of early repayment, rate is 1% or 0 (no charge)	+ 0	+ 1	+ 0	+ 1
Education level of borrower			− 1	
Borrower's company size by employed population		− 0		

Label = 0 means Repayments Success.

Label = 1 means Repayments Failure.

‘+’ means Supports, ‘−’ means contradicts.

**Figure 6:**
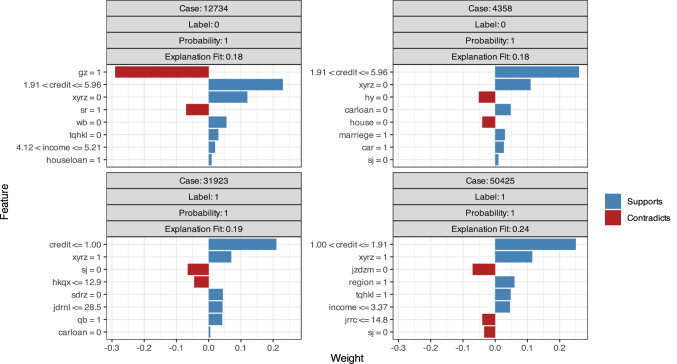
Variable weights of the RF model.

For some variables, such as credit, xyrz, sj, jzdzm and sdrz, the prediction results of RF (Fig. 6 and Table 5) are similar to those of GBM and NN. Notably, borrowers with job verification or income authentication and borrowers without marriage confirmation are more likely to fail to repay their loans. Job and income are important indicators of personal economic capacity, and more than 95 percent of borrowers pass the job and income verification required by the P2P lending company. Borrowers who are successfully financed without job authentication or income certification tend to borrow less money for a shorter period of time; as a result, those borrowers have lower default risk. Meanwhile, those borrowers are also under the supervision of the P2P lending company after borrowing, resulting in a higher probability of repayment. In contrast, borrowers who have passed job authentication or income certification borrow more for a longer period of time, resulting in greater repayment pressure and a lower successful repayment probability. The RF prediction results are slightly unexpected but are rational when considering the combined effect of the amount and duration of financing.

**Table 5:** Variable weights of the RF model.

Questions	Prediction weight			
	Predicting success		Predicting failure	
	Label = 0	Label = 1	Label = 0	Label = 1
Credit grade classified by Renrendai	+ (1.91,5.96]	+ (1,1.91]	+ (1.91,5.96]	+ (0,1]
Whether Renrendai has verified the borrower's information only online	+ 0	+ 1	+ 0	+ 1
Whether Renrendal has confirmed the borrower's information onsite				+ 0
Whether Renrendai has verified the borrower's mobile phone	+ 0	− 0		− 0
Whether Renrendai has verified the borrower's job			− 1	
Whether Renrendai has verified the borrower's income			− 1	
Whether Renrendai has verified the borrower's marriage	− 0			
Whether Renrendai has verified the borrower's residence		− 0		
Whether Renrendai has verified the borrower's microblog			+ 0	
Number of bidders in a project		− (0,14.8]		
Cost of early repayment, the rate is 1% or 0 (no charge)		+ 1	+ 0	− (0,12.9]
Borrower’s age				+ (0,28.5]
Borrower’s income by month in RMB		+ (0,3.37]	+ (4.12,5.21]	
Whether borrower possess a house	− 0			
Whether borrower possesses a car	+ 1			
Whether borrower possess a home loan			+ 1	
Whether borrower possess a car loan	+ 0			+ 0
Borrower’s city of residence		+ 1		
qb				+ 1

Label = 0 means Repayments Success.

Label = 1 means Repayments Failure.

‘+’ means Supports, ‘−’ means contradicts.

**Figure 7:**
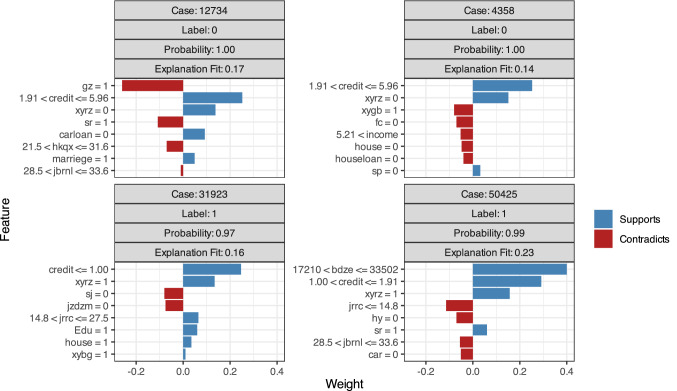
Variable weights of the XGBT model.

Based on the preceding studies, we use XGBT to examine the various factors that affect loan repayment. We confirm the conclusions of the previous methods (Fig. 7 and Table 6). A higher credit score, onsite certification and guarantees improve the probability of repayment. In contrast, verification of a borrower’s job, income, mobile phone, residence or marital status has a negative effect on repayment success. Interestingly, borrowers with higher income and loans with longer terms are associated with an increased probability of repayment failure. When a borrower’s income level is greater than 5, that is, when a borrower’s monthly income is greater than 10,000 RMB, they are less likely to repay successfully. This result is unexpected, but because a borrower with a higher monthly salary can borrow a larger amount over a longer period, the repayment amount would be far greater than his personal salary, which results in a higher probability of repayment failure.

**Table 6:** Variable weights of the XGBT model.

Questions	Prediction weight			
	Predicting success		Predicting failure	
	Label = 0	Label = 1	Label = 0	Label = 1
Credit grade classified by Renrendai	+ (1.81,5.65]	+ (1.00,1.91]	+ (1.91,5.65]	+ (0,1]
Whether Renrendai has verified the borrower's information only online	+ 0	+ 1	+ 0	+ 1
Whether Renrendal has confirmed the borrower's information video data	+ 0			
Whether Renrendai has verified the borrower's mobile phone				− 0
Whether Renrendai has verified the borrower's income		+ 1	− 1	
Whether Renrendai has verified the borrower's marriage		− 0		
Whether Renrendai has verified the borrower's residence				− 0
Whether Renrendai has verified the borrower's credit	− 1			+ 1
Number of bidders in a project		− (0,14.8]		+ (14.8,27.5]
Load amount borrowed		+ (17,210;33502]		
Time for repayment, in months			− (21.5,31.6]	
Education level of borrower				+ 1
Borrower’s age		− (28.5,33.6]	− (21.5,31.6]	
Whether borrower is married			+ 1	
Borrower’s income by month in RMB	− (0,5.21)			
Whether borrower possess a house	− 0			+ 1
Whether borrower possesses a car		− 0		
Whether borrower possess a home loan	− 0			
Whether borrower possess a car loan			+ 0	

Label = 0 means Repayments Success.

Label = 1 means Repayments Failure.

‘+’ means Supports, ‘−’ means contradicts.

### Model comparison

Financial regulatory organizations and P2P companies must be able to make reliable predictions of borrowers’ default to control financial risk and protect small investors, as China's P2P lending companies have suffered a large-scale collapse in the past three years. Additionally, machine learning methods, such as RF, GBM, XGBT and NN, have been demonstrated to be powerful in improving the capacity of risk prediction and mitigating information asymmetry. To facilitate our research, delay in repayment or repayment failure is arbitrarily recoded as “1” within our datasets. Then, several machine learning methods, including RF, XGBT, NN, and GBM, are implemented with 5-fold cross-validation on each classifier to measure the model performance in terms of prediction accuracy and kappa value. In addition, we included the traditional logistic regression, the generalized linear model (GLM), to compare the prediction results. Table 7 lists the accuracy and kappa value for each classification model.

**Table 7:** Prediction accuracy and kappa value of each classification model (%).

	RF	XGBT	GBM	NN	GLM
Accuracy	0.984	0.982	0.979	0.972	0.969
Kappa	0.967	0.964	0.958	0.944	0.938

*XGBT* extreme gradient boosting tree model, *GBM* gradient boosting model, *NN * neural network model, *GLM* generalized linear model.

**Figure 8:**
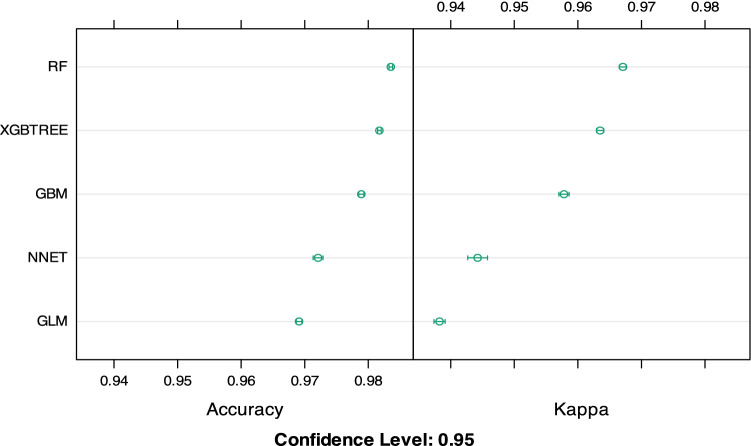
The prediction accuracy of the five models.

Table 7 and Fig. 8 show the accuracy and kappa value of the five classification models. RF tends to produce the best repayment default prediction for P2P borrowers on Chinese P2P lending platforms within the same attribute selection. The overall prediction accuracy of RF is 98.4%, and the kappa value is 96.7%, demonstrating that the RF model outperforms the other models in terms of the prediction accuracy of default risk. In addition, the accuracies of XGBT, NN, and GBM all exceed 90%, which indicates that all four methods show potential for accurately predicting default risk in the field of worldwide finance.

To further test the performance of the different models, the precision, recall, and F1 values for the five models were calculated, and the results are shown in Table 8 below. All of the models have achieved over 94% performance of each metric, and all are superior to our benchmark model (GLM). This further shows that the four models in this paper all have good effects on the prediction of default. The random forest model has the best performance, which is consistent with the highest accuracy and kappa in the previous test.

**Table 8:** Precision, recall and F1-score of each classification model (%).

	RF	XGBT	GBM	NN	GLM
Precision	0.998	0.996	0.967	0.995	0.942
Recall	0.971	0.969	0.991	0.941	0.948
F1-score	0.985	0.982	0.979	0.967	0.969

*XGBT* extreme gradient boosting tree model, *GBM* gradient boosting model, *NN * neural network model, *GLM* generalized linear model.

## Discussion

Borrowers’ defaulting on loans not only damages the interests of lenders and social trust but also represents financial risks. For the last three years, extensive effort in the academic field has concentrated on developing machine learning techniques that help regulators and P2P platforms carry out accurate and efficient prediction of large-scale overdue repayment or loan default. The main purpose of our paper is to utilize recently developed machine learning methods to devise credible and accurate prediction techniques to explore the key factors that affect P2P loan default. We extract data from one of the 10 largest P2P lending companies in China, Renrendai.com, which scholars have widely used for empirical evaluation of machine learning methods, to compare the performance of different forecasting models. In contrast to previous studies and designs, we emphasize the use of machine learning methods to predict factors that might affect the probability of loan defaults rather than simply comparing the strengths and weaknesses of models. Furthermore, we emphasize the verification status of borrowers’ personal information, especially nonmandatory verification information, such as mobile phones, videos and marital status, which has not received close attention in previous studies. To protect privacy, some of the borrowers’ information is not disclosed on the web page of the platform. We wield software to capture the background information and the machine learning method to predict borrowers’ repayment default, which is quite different from previous research.

Our empirical study also indicates that, in addition to credit rating, education level, marital status, social relations, appearance, text description and other factors investigated in previous empirical studies using traditional methods (OLS, logit or probit models), the borrower’s authentication information included in our datasets, such as verification of mobile phones, videos, jobs or income, has strong predictive effects on the probability of repayment. Specifically, we first explore the most important factors affecting repayment. GBM successively calculates the importance of each factor, namely, credit rating and online credit authentication, followed by mobile phone verification, video verification, and number of winning bidders in a project. The top 5 factors fully prove the importance of mobile phone verification and video verification in predicting borrowers' repayment ability. Furthermore, the GBM and NN results show that verification of a borrower’s video, mobile phone, job or education level has a positive effect on the probability of loan default, whereas authentication of a borrower’s identification and assets has a negative effect on the probability of loan default. Further investigation concerning the weights of relevant factors by GBM, NN and XGBT confirms that borrowers with mobile phone verification or residence certification are more likely to fail in repayment. The NN and XGBT results also indicate that a higher credit rating, onsite certification and guarantees improve the probability of repayment. Furthermore, RF and XGBT both indicate that borrowers with job verification or income authentication and borrowers without marriage confirmation are more likely to fail to repay their loans. In summary, all the empirical results show that borrowers who have passed video, mobile phone, job, residence or education level verification have a higher probability of default, whereas those who have passed identity or asset certification are less likely to default on loans.

Meanwhile, we tested the validity of our methods and techniques. Based on the indicators obtained by the K-fold cross-validation test, we conducted a t-test on the metrics of each model, namely, accuracy, kappa, precision, recall and the F1 score to test whether the RF model is the best model among all models. The hypothesis of the test is as follows: Hypothesis 0 represents the metrics of the RF and indicates there is no difference between its metrics and those of other models. Hypothesis 1 indicates that the RF metrics are better than those of other model metrics. The t-test results are shown in Table 9. The results show that the RF model performs well within the 95% confidence interval. Regarding accuracy, only the performance of XGBoost showed little difference from that of RF (P = 0.147), and the performance of the other models was significantly weaker than that of RF (P < 0.01). Similarly, in the recall metric, only the GBM model failed the t-test (P = 0.983), which shows that the performance of the GBM model in this metric is slightly different from or better than that of the random forest model. In addition, among kappa, precision and the F1 score, the RF model is better than other models (P < 0.01).

**Table 9:** T-test on metrics of random forest model and other models.

	XGBT	GBM	NN	GLM
Accuracy	p = 0.147	p < 0.001	p < 0.001	p < 0.001
Kappa	p = 0.003	p = 0.002	p < 0.001	p < 0.001
Precision	p = 0.007	p < 0.001	p = 0.004	p < 0.001
Recall	p = 0.008	p = 0.983	p < 0.001	p < 0.001
F1-score	p = 0.002	p < 0.001	p < 0.001	p < 0.001

*XGBT* extreme gradient boosting tree model, *GBM* gradient boosting model, *NN * neural network model, *GLM* generalized linear model.

p is the t-test p-value.

We further obtain the confidence intervals of the various metrics of the random forest through the nonparametric test method, the bootstrap method. We choose 1000 replacement samplings to re-estimate the mean and confidence interval, and the results are shown in Table 10 below. The mean values of all indicators passed the t-test (P < 0.01).

**Table 10:** Using the bootstrap method to obtain the confidence interval of each metric of the random forest.

	Mean	p_values	Lower_CI	Upper_CI
Accuracy	0.985	< 0.01	0.982	0.986
Kappa	0.969	< 0.01	0.968	0.972
Precision	0.998	< 0.01	0.996	0.999
Recall	0.971	< 0.01	0.97	0.974
F1-score	0.984	< 0.01	0.984	0.986

Compared with other studies, in traditional statistical models such as logit and probit models, the accuracy predicted by our model has been significantly improved, surpassing the probit model of Freedman et al.^4^ in 2008 and the Cox model of Lin et al. in 2013^8^. Moreover, our model also effectively recognizes the importance of each feature. Regarding machine learning models, such as SVM and NN models, our model accuracy was compared with the SVM and NN models used by Huang et al. in 2004^3^, and the accuracy of our model increased by approximately 10%. Compared with the SVM model^2^ used by Jin et al. in 2015, it increased by approximately 20%. This may be due to the small number of features in their dataset (20 features for Huang’s study and 10 for Jin’s study) and the smaller dataset (339 for Huang's dataset, while Jin's data are almost half of our dataset).

In summary, the random forest model trained in this study performs well in the classification task of whether default will occur. It can identify the default objects well, and through the feature importance analysis of the model, we can determine which features are important factors that affect the default rate. It has positive significance for the risk control of financial companies.

## Conclusion

Predicting the occurrences of loan default in a credit market such as a P2P lending platform is a crucial and challenging task. More accurate prediction models would be highly beneficial since the failure of a P2P lending platform could trigger a series of financial risks. Our empirical results confirm that mobile phone, video, marriage, income, job and other verification information play an important role in predicting borrowers' repayment ability. Furthermore, our findings show that machine learning methods have broad application prospects in the prediction of P2P loan default but also provide important techniques for regulatory agencies and P2P lending companies in terms of borrower screening and platform management, which could ultimately reduce the risk of online financial markets worldwide.

Although our research conclusions are based on the prediction of repayment default on P2P platforms, machine learning methods can also be widely used in borrowers' credit risk assessment to help banks and borrowers effectively resist financial risks. Especially in the current global coronavirus disease 2019 (COVID-19) context, the global economic downturn is obvious, financial risks are gathering, and the financial market has been seriously impacted, highlighting the efficiency and accuracy of machine learning methods. According to McKinsey's 2019 report, machine learning can reduce bank credit loss by 10% and reduce credit decision-making time by 25% to 50%. Zestfinance, which was among the first to apply machine learning methods to credit risk assessment, found that the performance of machine learning models was 40% higher than that of traditional credit assessment models. Therefore, our research methods and conclusions can be applied to the following fields.

First, our methods and conclusions can help banks establish the credit scoring model of enterprise borrowers. Our approach can help predict the borrower's credit score and repayment ability in combination with the borrower's asset status, credit record, profitability of existing business, business growth rate, turnover of working capital, industry development prospect, operation status of affiliated enterprises, and audit data of industrial and commercial and tax departments. We can first use the machine learning program to analyze and preprocess the relevant data of enterprise borrowers preliminarily and then use the random forest method or XGBoost algorithm to realize feature selection, split the data, construct the machine learning training model, and use the machine learning model to complete the data prediction. In the last stage, to measure the effect of the model, we can use various evaluation indicators, such as accuracy, kappa, precision and recall, to judge the prediction effect. In addition, we can also use machine learning methods to evaluate the credit status of individual borrowers. Combined with the data of individual borrowers' assets and credit records, social media such as WeChat or Facebook participation, network payment such as Alipay or Ebay, personal, cultural and religious background, social networks, and demographic characteristics such as gender, age, occupation, education, can potentially provide data that will be used to predict borrowers' credit score and repayment ability.

Second, our research methods and conclusions also have a good reference for financial regulators. First, regulators can use the big data of enterprises to predict regional financial risks and then take regulatory measures, such as reducing or increasing working capital, increasing or reducing the bank reserve ratio, and increasing or reducing the bank lending rate, to achieve the purpose of regulating financial risks. Second, regulators can use machine learning methods to identify financial fraud and present risk monitoring data to lenders through risk warnings. Specifically, regulators can extract the transaction data of credit cards, use machine learning methods for training and reverse testing, extract the key features of financial fraud, and distinguish these features from normal transactions. This identification mechanism helps regulatory authorities effectively identify financial fraud, especially those with poor business conditions, and take the opportunity for COVID-19 prevention and control to defraud government subsidies.

In addition, according to our research conclusions, we believe that the government can pay better attention to individual credit customers in the following ways to minimize financial disasters and recover from them. First, identify the borrower's borrowing demand and repayment ability. For individual borrowers who have borrowing needs but do not obtain loans in time, it is necessary to re-evaluate the borrower's career development prospects and social relationship networks by using a machine learning method and pay more attention to the future rather than credit records, assets, collateral and other past information. For borrowers with good development prospects, the government can issue policy subsidies to help borrowers overcome difficulties. Second, investment suggestions should be given to individual borrowers according to the risk acceptance and repayment ability of different borrowers. Investment channels such as the stock market, bond market, banks and internet finance have different forms of risk, terms, costs and returns. Third, financial institutions are encouraged to grant loans to individual borrowers with high credit ratings in the form of subsidies and preferential policies to financial institutions.

In general, our research methods and conclusions can be used for reference by banks, financial regulators, governments, corporate borrowers and individual borrowers. It is worth mentioning that our study does not consider the changes in macroeconomic factors and regulatory policies. In particular, great changes have occurred in China's economy and regulatory policies in the past five years. This is the deficiency of our research, which can be further explored in future studies.

## Data Availability

The data of this study are available from the authors upon request.
